# Preclinical Development of Tuspetinib for the Treatment of Acute Myeloid Leukemia

**DOI:** 10.1158/2767-9764.CRC-24-0258

**Published:** 2025-01-13

**Authors:** Himangshu Sonowal, William G. Rice, Raphael Bejar, Joo-Yun Byun, Seung Hyun Jung, Ranjeet Sinha, Stephen B. Howell

**Affiliations:** 1 Department of Medicine and the Moores Cancer Center, University of California, San Diego, San Diego, California.; 2 Aptose Biosciences, Inc., San Diego, California.; 3 Hanmi R&D Center, Seoul, Korea.

## Abstract

Tuspetinib (TUS) is a well-tolerated, once daily, oral kinase inhibitor in clinical development for treatment of acute myeloid leukemia (AML). Nonclinical studies show that TUS targets key prosurvival kinases with IC_50_ values in the low nmol/L range, including SYK, wild-type (WT) and mutant forms of FLT3, mutant but not WT forms of KIT, RSK2, and TAK1–TAB1 kinases, and indirectly suppresses expression of MCL1. Oral TUS markedly extended survival in subcutaneously and orthotopically inoculated xenograft models of FLT3-mutant human AML, was well tolerated, and delivered enhanced activity when combined with venetoclax (VEN) or 5-azacytidine. *In vitro*, TUS demonstrated potent killing of AML lines [concentration needed to reduce the growth of treated cells to half that of untreated cells (GI_50_) = 1.3–5.2 nmol/L] and Ba/F3 cells expressing WT (GI_50_ = 9.1 nmol/L) or various mutant forms of FLT3 (GI_50_ = 2.5–56 nmol/L). In AML lines, the multikinase targeting capacity of TUS suppressed phosphorylation of SYK, FLT3, STAT5, MEK, ERK, AKT, mTOR, 4E-BP1, and S6K kinases. Cells selected for stable acquired resistance to TUS exhibited increased BAX and hypersensitivity to VEN (1900 fold), navitoclax, and MCL1 inhibitors. MV-4-11 FLT3-ITD clones expressing NRAS^G12D^ revealed that high-level expression of NRAS^G12D^ generated modest resistance to TUS and greater resistance to VEN, yet the TUS/VEN combination exhibited synergy in the NRAS^G12D^ AML model. Favorable preclinical safety and pharmacology properties, the efficacy of the TUS/VEN combination in a murine model, and the synthetic lethal vulnerability to VEN that accompanies TUS resistance provide the basis for exploration of the TUS/VEN combination in patients with relapsed or refractory AML.

**Significance:**

This article reports preclinical development of TUS, an oral kinase inhibitor currently in clinical development for treatment of AML. The article covers the studies of TUS activities on cellular targets and the nonclinical studies that supported the advancement of TUS to a phase I/II trial of TUS/VEN in refractory AML and a phase I/II trial of TUS/VEN/5-azacytidine in newly diagnosed patients with AML (NCT03850574).

## Introduction

Acute myeloid leukemia (AML) is a highly heterogeneous and aggressive hematologic malignancy in which hematopoietic stem cells proliferate excessively due to dysregulation of prosurvival signaling and death mechanisms. Recurrent genetic abnormalities frequently occur in the FLT3, DNMT3A, IDH1/2, MLL, NPM1, KIT, RAS, and TP53 genes, along with other adverse genetic, epigenetic, and metabolic alterations. Moreover, extraordinary plasticity among transcription and phenotypic states enables rapid emergence of resistance to therapeutics (1–3). Treatment of patients with AML with approved FLT3 inhibitors (gilteritinib, quizartinib, midostaurin, and sorafenib), IDH inhibitors, the BCL2 inhibitor venetoclax (VEN), chemotherapeutics, and hypomethylating agents is linked to a myriad of resistance mutations, and no drug has been developed with a broad range of activity and safety across patients with AML with a diversity of adverse mutations (4–10).

Tuspetinib (TUS) is an oral noncovalent multikinase inhibitor designed to simultaneously target a select set of kinases that drive prosurvival pathways operative in myeloid malignancies and that enable rescue pathways to engender drug resistance. Such targets include the SYK, wild-type (WT) and mutant forms of FLT3, mutant but not WT forms of KIT, JAK1/2, RSK2, and TAK1–TAB1 kinases, and TUS indirectly suppresses expression of the MCL1 antiapoptotic protein. Simultaneous suppression of the circumscribed set of targets distinguishes TUS from currently approved AML drugs. We report here on TUS activities on cellular targets and the nonclinical studies that supported the advancement of TUS to a phase Ia/b trial in patients with AML who are resistant, refractory, or intolerant to established therapies.

## Materials and Methods

### Drugs and antibodies

TUS (HM43239) was provided by Hanmi Pharmaceutical and Aptose Biosciences. The stock solution was prepared by dissolving TUS in 100% DMSO to a final concentration of 10 mmol/L. All other drugs and antibodies used for Western blotting were purchased from commercial sources.

### Kinase screens

Potency screens against recombinant forms of kinases were performed by Thermo Fisher Scientific and Reaction Biology, Inc. Assays of the binding of TUS to WT and mutant forms of FLT3 were performed by DiscoverX. Dissociation constants (K_d_) were calculated from two independent experiments performed in duplicate using a concentration range of 0.002 to 100 nmol/L (11 points, 1/3 serial dilution). Data from these screens are available on request.

### Cell lines and growth inhibition assays

MV-4-11 (CRL-9591), HS-5 (CRL-3611), and KG1a (CCL-246.1) cells were obtained from ATCC. MOLM-13 (ACC 554) and MOLM-14 (ACC 777) were obtained from DSMZ (Deutsche Sammlung von Mikroorganismen und Zellkulturen). MOLM-14 cells contain one WT FLT3 allele and one FLT3-ITD allele. MOLM-14 FLT3-ITD/F691 cells were prepared by transfection of MOLM-14 cells with a vector expressing the FLT3 TKD F691L mutation. MV-4-11 cells expressing NRAS^G12D^ were prepared by transduction with a lentiviral vector encoding the NRAS^G12D^ mutation. Assays using a panel of parental Ba/F3 into which an additional copy of either WT or a mutant form of FLT3 was introduced were performed at Kyinno Biotechnology Co., Ltd. All cell lines were short tandem repeat authenticated at ATCC or at DSMZ when originally obtained, and no further authentication was done after receipt of the cells in the laboratory.

To establish TUS-resistant (TUS/R) MOLM-14 sublines, 1 × 10^6^ cells/mL were grown in medium supplemented with 10 ng/mL FGF2 and increasing concentrations of TUS. The medium was replaced every 2 to 3 days with addition of TUS and FGF2; viability was determined by trypan blue exclusion. TUS/R sublines capable of sustained growth in media containing 75 nmol/L TUS were obtained after a 3-month period.

### Immunoblotting

Cells were treated as described in respective Results sections and then lysed with RIPA buffer. Western blot analyses were performed with antibodies capable of detecting both total and phosphorylated forms of each kinase purchased from commercial sources. Information on the antibodies used is provided in the Supplementary Materials section.

### Generation of luciferase/GFP expressing cells for orthotopic studies

Stable MOLM-14-Luc/GFP cells were generated by transfecting MOLM-14 cells with CMV-Luciferase (firefly)-2A-GFP (Neo; GenTarget Inc.) followed by selection in 1 μg/mL puromycin. MV-4-11 Luc and MOLM-13 Luc cells were generated by transfecting CMV-Firefly luciferase lentivirus (Cellomics Inc.) followed by selection in G418.

### HS-5 coculture and fibrinogen and IgG stimulation

HS-5 cells were obtained from ATCC. The HS-5 stromal cells (5 × 10^5^ cells/well) were seeded into six-well plates, and 24 hours later, MOLM-14 cells (1 × 10^6^ cells/well) were seeded into the same plate and then cultured for 48 hours. Cultures were then exposed to test drugs for 2 hours, harvested, and lysates were prepared for Western blotting. For stimulation studies, MOLM-14 or KG-1a cells were seeded at 5.0 to 6.0 × 10^6^ cells/well into six-well plates in 2 mL medium. Cells were stimulated with 500 μg/mL fibrinogen (Sigma-Aldrich, Catalog # F3879) for 1 hour at 37°C. Alternatively, they were treated with 10 μg/mL IgG (Invitrogen, Catalog # 31154) for 10 minutes on ice followed by IgG cross-linking with 1 μg/mL anti–human IgG antibody for 10 minutes on ice. After these incubations, the media were removed and the cells were washed with cold PBS, lysed, and analyzed by Western blotting.

### Assay using Ba/F3 cells

Assays using Ba/F3 cells expressing WT and mutant forms of FLT3 were performed by Kyinno Biotechnology. The cells were cultured in the medium recommended by the source for each cell line. Growth inhibition assays were performed using either Cell Titer 96 Aqueous One Solution Cell Proliferation assay (Promega, # G3581) or CCK8 (Dojindo, # CK-04). Data presented are mean ± SEM from a minimum of three independent experiments each performed with triplicate cultures for each drug concentration and a drug exposure of 72 hours unless otherwise stated. IC_50_ values were determined from growth inhibition curves fitted using GraphPad Prism and a nonlinear regression model with sigmoidal dose response. Unless otherwise indicated, data are presented as mean ± SEM.

### Bone marrow analysis

Immunoblotting and IHC analyses were performed with antibodies capable of detecting both total and phosphorylated forms of each kinase purchased from commercial sources. For assessment of p-FLT3, p-SYK, and p-ERK expression, femoral marrow harvested from control or TUS-treated mice was fixed and stained with primary antibodies to each phosphokinase.

### Genomic sequencing

Genomic DNA was isolated using QIAamp DNA Mini kit following the manufacturer’s protocol. High-throughput DNA sequencing was performed by Admera Health on parental MOLM-14 and each of the four TUS/R MOLM-14 lines.

Sequencing was performed on the NovaSeq X Plus 10B sequencer targeting at least 52 mol/L PE reads per sample (26 mol/L reads each side). Raw sequencing reads were quality-trimmed using Trim Galore (version 0.6.10), a wrapper for Cutadapt (version 4.6), to remove low-quality bases and adapter sequences. Following trimming, sequence alignment and somatic variant detection were performed using the Illumina Dragen Somatic Suite (version 4.3.6), with the human reference genome GRCh38. The target regions for variant detection were defined using the Target BED file provided by Twist Bioscience.

Somatic variant detection was conducted in both tumor-only mode, in which all samples were treated as tumors, and paired mode, in which the parental MOLM-14 served as the normal control. The resulting variant call format files were annotated using the Ensembl Variant Effect Predictor (version 111) with the GRCh38.111 annotation set. Variants shared by all four lines that were predicted to alter protein coding sequences or impair proper splicing of exons were retained as putative mutations associated with TUS resistance.

Confirmation of the NRAS G12C mutation in the resistant lines (and its absence in the parental line) was performed using Qiagen’s AllTaq PCR Core Kit (250 U, #203123) to amplify NRAS exon 2 in the MOLM-14 parental and TUS/R lines with the specific forward and reverse primers:

NRAS G12C forward primer: ATG​TGG​CTC​GCC​AAT​TAA​CC
NRAS G12C reverse primer: TCC​GAC​AAG​TGA​GAG​ACA​GGA

Sanger sequencing using the PCR primers as sequencing primers was performed using Genewiz (Azenta Life Sciences) and analyzed using Geospiza software.

### *In vivo* models of AML

For subcutaneous inoculation, MV-4-11 and MOLM-14 FLT3-ITD/F691L cells were mixed with BD Matrigel Matrix (1:0.5 ratio), and 2 to 5 × 10^6^ cells in 0.15 mL were inoculated subcutaneously in NOD/SCID mice. Tumor size was assessed using a Vernier caliper 2 to 3 times weekly and volume was calculated using the formula for an ellipsoid sphere. For orthotopic inoculation, MOLM-14-Luc/GFP cells (2 × 10^6^ cells in 0.03 mL PBS) or MV-4-11-Luc and MOLM-13-Luc cells (1 × 10^6^ cells in 50 μL Hank’s balanced salt solution) were injected into the left tibia of NOD/SCID mice. Tumor growth was monitored weekly by bioluminescence imaging using an IVIS Lumina Series III *In vivo* imaging system (PerkinElmer Inc.). The Institutional Animal Care and Use Committee of the Hanmi Research Center approved the animal study protocol.

### *In vivo* study group assignment

For both subcutaneous and bone marrow orthotopic models, after confirming tumor formation by palpation or bioluminescence imaging, mice were randomly assigned into experimental groups.

### Statistical analysis

The effect of treatment with TUS on survival was assessed using Kaplan–Meier plots that were analyzed with the log-rank (Mantel–Cox) test using GraphPad Prism. The effect of TUS treatment on subcutaneous tumor size was analyzed using one-way ANOVA followed by the Tukey test or two-way ANOVA followed by the Sidak test. A *P* value less than 0.05 was considered statistically significant.

### Data availability

Sequencing data are available under the NCBI BioProject accession number PRJNA1189604. Other data generated in this study are available upon request from the corresponding author.

## Results

### TUS inhibits WT and mutant forms of FLT3 and clusters of kinases active in AML

**Figure 1:**
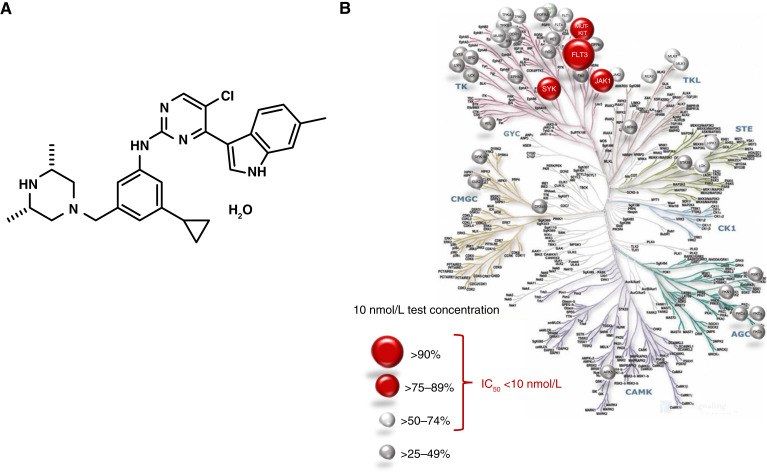
Structure of TUS and pattern of kinase inhibition. **A,** Structure of TUS. **B,** Principal kinases inhibited by TUS as determined from broad kinase screens.

TUS (Fig. 1A) is a small-molecule, noncovalent inhibitor of a select set of kinases. TUS was selected from a chemical library based on its selectivity and potency against a panel of defined kinases. Broad kinase inhibition screens documented its ability to inhibit kinases known to support proliferation of AML cells including SYK (IC_50_ = 2.9 nmol/L), WT and mutant forms of FLT3 (IC_50_ = 1.0–1.8 nmol/L), KIT-MUT (IC_50_ = 3.5–3.6 nmol/L), TAK1 (IC_50_ = 7 nmol/L), JAK1(IC_50_ = 2.9 nmol/L), JAK2 (IC_50_ = 6.3 nmol/L), RET, KDR, NTRK2, CLK2, LYN B, MLK1, and RSK-2 (IC_50_ = 9.7 nmol/L; Fig. 1B). Because different forms of FLT3 are important drivers of AML, we tested TUS in a biochemical enzyme activity screening assay against the various forms of FLT3. TUS inhibited FLT3 WT (IC_50_ 1.1 nmol/L), FLT3-ITD (1.8 nmol/L), and FLT3 D835Y (1.0 nmol/L). The differences in the potency of TUS between these forms of FLT3 were found to be similar to those of gilteritinib and midostaurin (Supplementary Table S1). Affinity studies showed that TUS potently bound to WT FLT3 (K_d_ = 0.58 nmol/L) and mutant forms of FLT3 (K_d_ range 0.2–1.3 nmol/L). In comparison, TUS K_d_ values indicated higher affinity than those of midostaurin and quizartinib (Supplementary Table S2).

The ability of TUS to kill cells with clinically relevant forms of FLT3 (WT, ITD, D835Y, ITD/F691L, and ITD/D835Y) was tested in a panel of murine Ba/F3 cells in which proliferation was dependent on these kinases. Cell-killing assays of Ba/F3 cells with FLT3 WT, ITD, and D835Y demonstrated concentrations needed to reduce the growth of treated cells to half that of untreated cells (GI_50_) for TUS <9.1 nmol/L, and TUS retained potency against the clinically relevant and dual mutant FLT3 ITD/F691L and ITD/D835Y forms with GI_50_ values of 56 and 16 nmol/L (Supplementary Table S3). Consistent with these observations, TUS exhibited GI_50_ values between 1.3 and 5.2 nmol/L when tested against three different AML cell lines expressing FLT3 ITD (MV-4-11, MOLM-13, and MOLM-14; Supplementary Table S4). Although *in vitro* binding affinity of TUS to WT and mutant forms of FLT3 was lower compared with quizartinib, the GI_50_ values of quizartinib were lower in the cell lines tested (Supplementary Tables S2 and S4).

**Figure 2:**
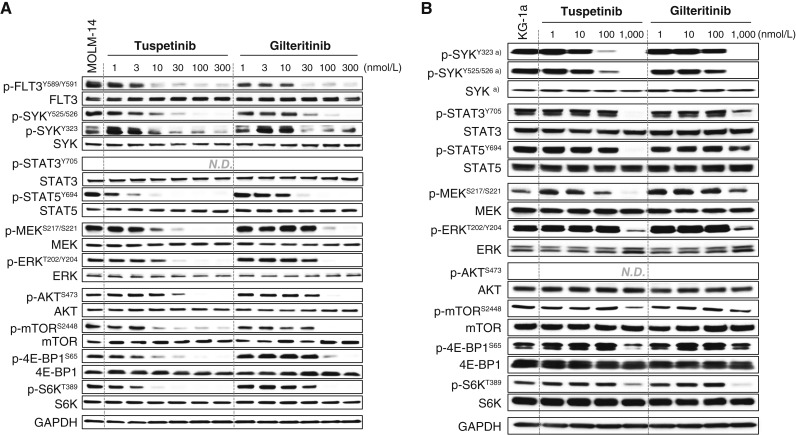
Relative effects of TUS and gilteritinib on phosphorylation of FLT3 and downstream kinases in (**A**) MOLM-14 and (**B**) KG-1a cells. A representative Western blot from two independent experiments is shown. **A** was assembled from several separate blocks.

Although FLT3 is an important target in AML, it is also essential to simultaneously target additional kinases that drive prosurvival pathways. Western blot analysis was used to assess the pharmacodynamic effects of TUS in the KG1a cell line in which FLT3 is WT and in the MOLM-14 cell line that contains a FLT3-ITD mutation. In both cell types, TUS reduced the ratio of p-FLT3 (Y589/Y591) to FLT3 at concentrations below 10 nmol/L and the ratio of p-SYK (Y525/526) to total SYK at concentrations below 30 nmol/L (Fig. 2A and B). TUS also reduced the phosphorylation of STAT5, MEK, ERK1/2, AKT, mTOR, 4EBP1, and S6K over the same low nmol/L concentration range. The comparison of TUS to gilteritinib demonstrated a similar pattern of kinase inhibition, with TUS being somewhat more potent at reducing phosphorylation of FLT3, STAT5, MEK, ERK1/2, mTOR, 4E-BP1, and S6K consistent with greater potency of TUS on the kinases of the downstream effector part of the RAS pathway.

### TUS dampens stroma-induced activation of FLT3-ITD signaling in AML cells

Signals from the bone marrow microenvironment (cytokines, growth factors, cell–cell contact etc.) are known to confer protection for AML cells against kinase inhibitors (11, 12). The impact of direct contact with cocultured human stromal cell line HS-5, a condition that mimics the marrow microenvironment, was assessed for TUS relative to gilteritinib using MOLM-14 cells. Compared with when the MOLM-14 cells were grown in the absence of HS-5 cells, when cocultured with HS-5 cells, the expression and basal p-FLT3 level was significantly reduced, whereas the basal and phosphorylated forms of SYK, JAK1, JAK2, STAT3, and STAT5 were markedly increased (Supplementary Fig. S1). TUS more potently reduced p-FLT3 than gilteritinib when cocultured with HS-5 cells, and it was 15-fold more potent in reducing p-SYK. Neither TUS nor gilteritinib inhibited p-JAK1, p-JAK2, p-STAT3, or p-STAT5 at concentrations <1 μmol/L. Thus, TUS retained its ability to suppress the FLT3- and SYK-driven pathways to a greater extent than gilteritinib in this marrow environment model.

Although SYK phosphorylation and activity is induced by multiple upstream signaling pathways, including FLT3, it was previously reported that the activation of FcγRI and integrin αIIbβ3 could also activate SYK (13). Activation of SYK by such alternative receptors contributes to maintenance of leukemia stem cells (14, 15). Testing the ability of TUS to modulate IgG- and fibrinogen-induced activation of the FLT3/SYK signaling pathway revealed that TUS was 2.1 to 15-fold and 4.5 to 13-fold more potent than gilteritinib at blocking fibrinogen- and immunoglobulin-mediated activation of SYK in KG-1a cells, respectively (Supplementary Fig. S2A and S2B).

### Pharmacology and toxicology of TUS

The pharmacology and toxicokinetics of TUS were initially evaluated in mice and supplemented with studies in rats and dogs. Following a single intravenous dose of 3 mg/kg TUS in male mice, the terminal plasma half-life was 5.5 hours and the *V*_ss_ was 2.4 L/kg, indicating a substantial extravascular distribution. Clearance was 0.3 L/hours/kg which was lower than hepatic blood flow. Following oral administration of TUS at doses of 1, 3, 10, and 30 mg/kg, the bioavailability ranged from 21.4% to 47.2%; the rate of oral absorption was slow to moderate with *T*_max_ occurring at 1.0 to 4.0 hours (Supplementary Fig. S3A). Systemic exposure increased with dose in a greater than dose proportional manner over the dose ranges from 1 to 30 mg/kg, an effect not observed in dogs. Plasma and tissue concentrations were measured in mice after single oral administration of 10 mg/kg. Following an oral dose of 10 mg/kg, tissue/plasma ratios ranged from 0.03 (brain) to 21.3 (lung; Supplementary Fig. S3B). The highest AUC_last_ values were for the lung, kidney, small intestine, and liver; tissue to plasma ratios were >11.7 for these tissues (Supplementary Fig. S3C). TUS pharmacokinetics in the dog differed in that bioavailability was much higher (99%) and AUC was linearly proportional to dose.

TUS was found to be metabolized by human liver microsomes and excreted primarily via the feces. Plasma protein binding of TUS was high in all species evaluated including mouse, rat, dog, and human plasma. TUS inhibited CYP2C8, CYP2C9, and CYP3A (Ki < 1 μmol/L) and exhibited irreversible inhibition of CYP1A2 with an IC_50_ shift of more than 2.1 fold.

The toxicity of TUS was characterized in repeat daily dose studies up to 4 weeks mainly in rats and dogs. In rats, the main target organ of toxicity was the lymphoid organs; in dogs, it was leucocyte depletion, decreased thymus and spleen weights, and tremors. In both species, all adverse events were reversible. There were no alterations in clinical chemistry parameters. Neuropharmacology and respiratory function assessment in rats disclosed no concerns. No treatment-related cardiovascular effects were observed in single dose and 4-week dog toxicology studies.

### TUS prolongs survival in multiple AML models

**Figure 3:**
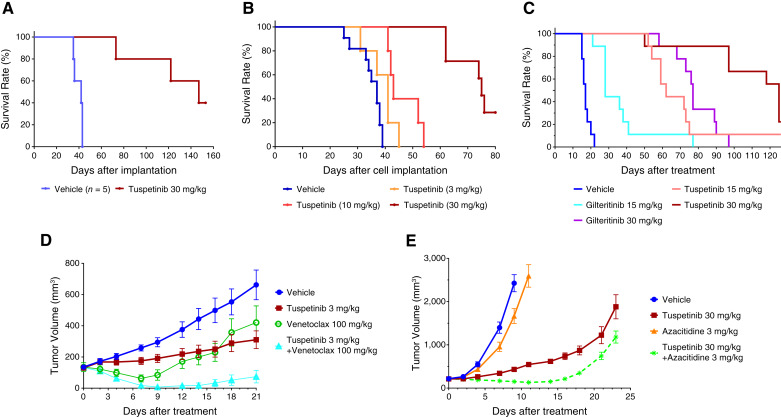
Effect of TUS alone on survival of mice orthotopically inoculated with FLT3-ITD-mutant AML cell lines; (**A**) MV-4-11; (**B**) MOLM-13; and, (**C**) MOLM-14. Efficacy of TUS in combination with VEN or AZA in mice inoculated subcutaneously with AML cell lines; (**D**) MV-4-11 tumors treated with either TUS (orally, every day), VEX (orally, every day), or the combination; (**E**) MOLM-14 FLT3-ITD/F691L tumors treated with either TUS, AZA (3 mg/kg i.v. days 0–4), or the combination. Data are presented as mean ± SEM.

The efficacy of single agent TUS was documented in NOD/SCID mice in which three different types of FLT-ITD AML cells (MV-4-11, MOLM-13, and MOLM-14) were inoculated orthotopically in the marrow. Oral daily administration of TUS 30 mg/kg every day markedly prolonged survival in all three models with clear dose dependency for MOLM-13. In the MV-4-11 model, the median survival was increased from 42 days for mice receiving vehicle to 147 days in TUS-treated mice (*P* = 0.0027; Fig. 3A). The increase in the MOLM-13 model was from 37 to 75 days (*P* < 0.0001; Fig. 3B) and in the MOLM-14 model, it was from 17 to 65 days (Fig. 3C). TUS reduced the percent of MOLM-14 cells in the marrow as a function of dose over the range of 15 to 45 mg/kg (Supplementary Fig. S4A). PIA assay documented marked reduction in pFLT3^Y589/591^ and p-SYK^Y525/526^ at a dose of 15 mg/kg (Supplementary Fig. S4B), and IHC analysis of marrow demonstrated reduced levels of p-FLT3, p-SYK, and p-ERK at a dose of 30 mg/kg (Supplementary Fig. S4C–S4E). Daily dosing of TUS at 30 mg/kg was well tolerated in mice with no significant weight loss in all three models.

### Combination therapy with TUS

Clinical studies indicate that response rates to FLT3 inhibitors can be improved by concurrent treatment with VEN (16, 17). An *in vitro* examination of the interaction of TUS and VEN was carried out using MV-4-11 FLT3-ITD cells (Supplementary Fig. S5). Synergy was quantified using Synergy Finder (18). Synergy Finder scores ranged from 12.6 to 29.1 across the four algorithms which indicate synergistic interaction at the cellular level. The score using the ZIP algorithm, which is considered the most predictive of synergy (19), was 12.6 ± 1.2. Although this score suggests only modest *in vitro* synergy, the efficacy of low-dose TUS was markedly enhanced by concurrent treatment with VEN when tested *in vivo* using the subcutaneous MV-4-11 model (Fig. 3D). *In vivo* testing was also carried out for the combination of TUS with 5-azacytidine (AZA) with similar beneficial results (Fig. 3E). Thus, despite having only modest synergy at the cellular level, the addition of either VEN or AZA to TUS had a beneficial impact in the xenograft models.

### Acquired resistance to TUS

**Figure 4:**
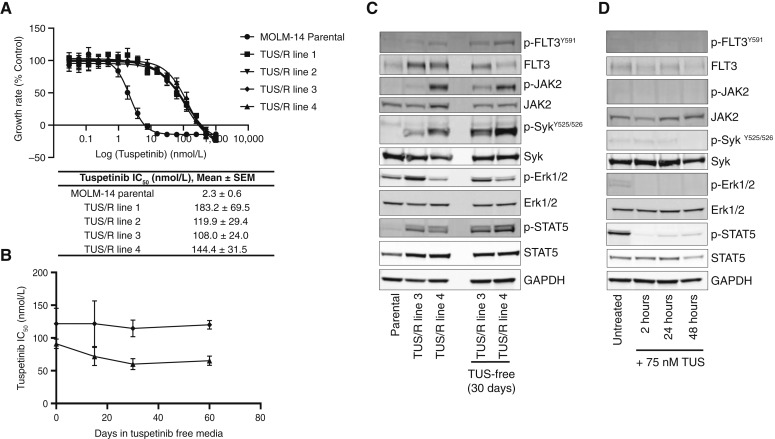
Characterization of TUS/R MOLM-14 cell lines. **A,** Effect of TUS on the growth rate of parental MOLM-14 cells and four sublines selected for growth in 75 nmol/L TUS (*P* < 0.01 vs. MOLM-14 parental cells for all TUS/R lines). **B,** Change in TUS IC_50_ of resistant cells when grown in TUS-free media. Data are presented as mean ± SEM from three-independent experiments. Western blot analysis documenting levels of selected kinases in (**C**) TUS-resistant lines 3 and 4 (**D**) after acute exposure to 75 nmol/L TUS for the indicated time. Representative data from two Western blots are shown.

Acquired resistance to kinase inhibitors is one of the primary reasons for therapeutic failure. Four TUS/R sublines were developed by exposing MOLM-14 continuously to successively higher TUS concentrations over a 3-month period. At the point when the cells with acquired resistance grew well in 75 nmol/L TUS, they were ∼60-fold resistant to TUS (IC_50_ 2.3 ± 0.6 vs. 138.9 ± 38.6 nmol/L; Fig. 4A), and resistance was stable 60 days after TUS removal (Fig. 4B). Western blot analysis of the resistant sublines growing in 75 nmol/L TUS, a concentration readily achieved in patient plasma, showed that p-FLT3 (Y591) was not reduced and that p-JAK2, p-SYK, p-ERK1/2, and p-STAT5 levels were all elevated relative to the total protein level of each kinase when compared with parental MOLM-14 cells. This activation of kinases was relatively stable when the cells were grown for 30 days in TUS-free media (Fig. 4C). In the parental MOLM-14 cells, although p-FLT3^Y591^ and p-JAK2 were expressed at levels too low to be evaluated, p-SYK^Y525/526^ and p-ERK1//2 were inhibited by 75 nmol/L TUS by 48 hours (Fig. 4D). Levels of cleaved PARP and cleaved caspase 3, makers associated with cell death, were lower in the resistant cells during growth in 75 nmol/L TUS (Supplementary Fig. S6).

We performed whole-exome sequencing analysis of the parental and all four TUS/R MOLM-14 cells. No additional changes were detected in the coding sequence of the FLT3-ITD already present in the parental MOLM-14 cells. However, the sequencing disclosed that an NRAS^G12C^ point mutation was present in all four resistant clones but not in the parental MOLM-14 cells. This was confirmed by sequencing of a PCR product covering the relevant section of the coding domain. Mutations in all four clones were also found in the 39 other genes listed in Supplementary Table S5 that play roles in transporter and metabolism pathways not known to be linked to FLT3 inhibitor resistance. The NRAS^G12C^ mutation has been linked to resistance to gilteritinib (8), but whether it alone mediates the changes observed in the TUS/R cells is currently unknown.

### TUS/R MOLM-14 cells are markedly hypersensitive to VEN

The TUS/R MOLM-14 cells were tested against a panel of drugs to determine cross-resistance or cross-sensitivity to other AML drugs. Interestingly, the TUS/R cells were cross-resistant to gilteritinib (∼14 fold) but remained completely sensitive to quizartinib (Supplementary Table S6). Although Western blot analysis showed constitutive activation of SYK, JAK, and PI3K in the TUS/R cells, they were not more sensitive to fostamatinib (SYK inhibitor), ruxolitinib (JAK inhibitor), or dactolisib (PI3K inhibitor); however, they were ∼1.9-fold hypersensitive to the MEK inhibitor trametinib.

**Figure 5:**
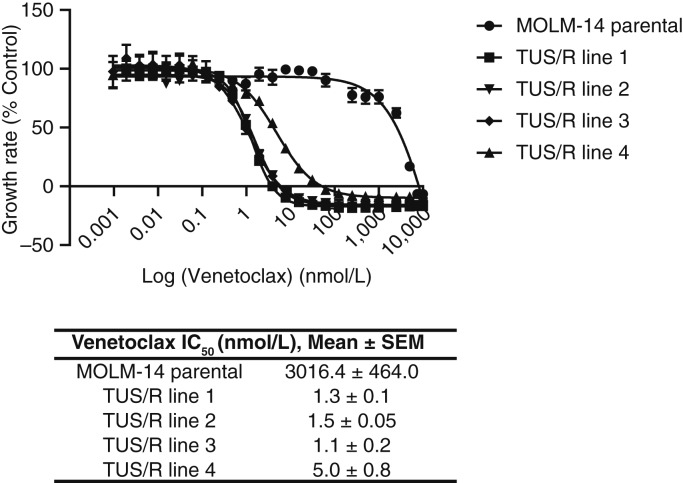
Resistance to TUS creates a synthetic lethal hypersensitivity to VEN. Growth inhibition curves documenting the effect of VEN on parental MOLM-14 and four sublines selected for growth in 75 nmol/L TUS. Data are presented as mean ± SEM from three-independent experiments; *P* < 0.001 vs. MOLM-14 parental cells for all 4 TUS/R lines.

Testing the sensitivity of TUS/R MOLM-14 cells to BCL2 and MCL1 led to unanticipated findings. The TUS/R cells were hypersensitive to BCL2 inhibitor navitoclax (∼27 fold) and even more hypersensitive to VEN (1,919 ± 927 fold). (Fig. 5; Supplementary Table S6). There was no increase in sensitivity to obatoclax, which simultaneously inhibits BCL2, BCL-w, BCL-xL, and MCL1. When inhibitors of MCL1 were tested, a muted synthetic vulnerability was found to extend to AZD5991 (∼8-fold hypersensitive) and S63845 (∼20-fold hypersensitive). Thus, acquisition of resistance to TUS created a substantial synthetic lethal vulnerability to these BCL2 inhibitors.

Given that the TUS/R cells exhibited such marked changes in sensitivity to drugs that target antiapoptotic proteins, the acute effects of 75 nmol/L TUS on major pro- and antiapoptotic protein levels in the parental MOLM-14 cells were compared with the changes found in the TUS/R sublines growing in the same concentration of drug. Acute exposure of parental MOLM-14 cells to TUS produced little change in BCL2 or BAX levels but reduced MCL1 by 70% and markedly increased BIM by 10 fold after a 48-hour exposure (Supplementary Fig S7). This was accompanied by large increases in cleaved caspase 3 and PARP (Supplementary Fig. S6). Relative to untreated parental cells, the TUS/R cells growing in 75 nmol/L TUS exhibited no change in BCL2 or MCL1, a small increase in BIM, but a very large increase in BAX. The observation that the TUS/R cells are markedly hypersensitive to VEN, and the fact that BCL2 is the target of VEN, indicate that under these circumstances the TUS/R cells are highly dependent on BCL2 for their survival.

### Effect of NRAS^G12D^ expression on sensitivity to TUS

**Figure 6:**
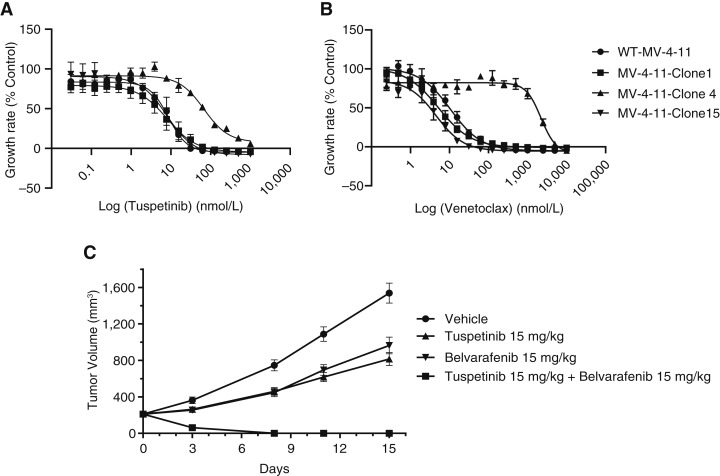
Effect of NRAS^G12D^ expression in MV-4-11 clones on sensitivity to (**A**) TUS and (**B**) VEN (data are mean ± SEM from three-independent experiments). **C,** Antitumor activity of TUS, belvarafenib, and the combination of both in mice bearing MV-4-11 NRAS^G12D^ subcutaneous tumors (drugs were given once daily for 15 days via oral gavage; data are presented as mean ± SEM).

The presence of the NRAS^G12D^ mutation is a strong negative prognostic indicator in patients with AML. MV-4-11 cells were engineered to express NRAS^G12D^ and three clones (clones 1, 4, and 15) with different levels of expression of NRAS^G12D^ were analyzed for their sensitivity to TUS (Supplementary Fig. S8A–S8D). A 13 to 14-fold increase in levels of NRAS^G12D^ expression in clones 1 and 15 did not alter sensitivity to TUS or to VEN. In contrast, clone 4 expressed much higher levels of NRAS^G12D^ (∼38 fold) than clone 1 and clone 15; clone 4 with high NRAS^G12D^ expression was 7.6-fold resistant to TUS and 214-fold resistant to VEN (Fig. 6A and B). Clone 4 with high NRAS^G12D^ expression had a 21-fold loss of sensitivity to gilteritinib and a 3.8-fold loss of sensitivity to quizartinib (Supplementary Table S7). All three clones of NRAS^G12D^-expressing cells were more sensitive to Raf and MEK inhibition by belvarafenib (3.6–7.4 fold) and trametinib (2.5–5.0 fold), respectively (Supplementary Table S7). The sensitivity to belvarafenib was independent of the NRAS^G12D^ expression level. Sensitivity to MCL1 inhibitors S63845 and AZD5991 correlated with NRAS^G12D^ expression in clones 1 and 15; conversely, the higher level NRAS^G12D^ expression in clone 4 was associated with 1.3 to 1.5-fold loss of sensitivity to these MCL1 inhibitors (Supplementary Table S7). Thus, the level of expression of the mutant NRAS^G12D^ rather than just the presence of the G12D mutation seems to be the most important determinant of resistance and sensitivity.

An *in vitro* examination of the combination of TUS with belvarafenib, S63845, or VEN was performed using the MV-4-11 cells expressing the highest levels of NRAS^G12D^ (clone 4). Analysis of synergy scores for combinations of TUS and these drugs demonstrated a strong enhancement of activity across all four algorithms for each combination (Supplementary Table S8). Interestingly, the *in vitro* combination of belvarafenib with TUS at the cellular level demonstrated synergy scores reflective of additive to slightly synergistic activity. When the combination of belvarafenib with TUS was further investigated *in vivo* with a subcutaneous xenograft model, the treatment completely eliminated the NRAS^G12D^-expressing MV-4-11 tumors (Fig. 6C). Together, these data suggest that TUS combines effectively with a variety of other classes of antileukemic agents and that such combinations may be useful for the treatment of patients with AML with RAS mutations and potentially other difficult-to-treat adverse mutations.

## Discussion

AML is a highly heterogeneous disease among patients and within a single patient, and the hyperproliferation and survival of AML cells is influenced by a multitude of cell surface and intracellular mediators. Because mutations in the FLT3 receptor represent the most common type of mutation in AML, much attention has been placed on the use of kinase inhibitors to suppress the FLT3 pathway. AML clones driven primarily by mutant FLT3 require a therapeutic agent that sustains near-complete inhibition of the FLT3 enzymatic activity and suppresses bypass pathways that can sustain prosurvival signaling even in the absence of FLT3 activity. To complicate matters further, the common presence of other oncogenic drivers in AML dictates the need to target additional pathways (8, 20–22). TUS simultaneously targets both WT and clinically significant mutant forms of FLT3, as well as a spectrum of other kinases that participate in rescue pathways that support survival and mediate resistance to various agents. Screens using recombinant kinases pointed to a unique pattern of inhibition across the kinome with sub- or low-nanomolar potency for FLT3, SYK, mutant forms of KIT, TAK1, JAK1, and JAK2 of the JAK/STAT pathway, RET and RSK of the RAS/MAPK pathway, and KDR, NTRK2, CLK2, LYN B, and MLK1 kinases, many of which have been implicated in drug resistance pathways. Thus, TUS offers the possibility of simultaneously inhibiting a set of kinases that collectively drive survival pathways and impart resistance to a variety of other drugs through rescue pathways.

Testing in Ba/F3 cells engineered to express additional copies of WT and the most clinically relevant mutant FLT3 forms demonstrated that TUS inhibits both WT and mutant forms of FLT3 (including the clinically important FLT3-ITD, FLT3-ITD/F691L, FLT3 D835Y, and FLT3-ITD/835Y forms). But TUS is more than a FLT3 inhibitor and it also suppressed the phosphorylation of SYK, STAT5, MEK, ERK, AKT, mTOR, 4E-BP1, and S6K kinases in cells, thereby simultaneously targeting multiple survival- and resistance-enabling pathways. TUS also retained low nmol/L potency when cells were grown in a culture system that mimics to some extent the normal marrow environment in which leukemic cells are protected by stromal growth factors and cytokines. SYK is a critical mediator of the stromal signaling that sustains AML cells (15, 23, 24); thus, potent inhibition by TUS offers the potential of offsetting this resistance mechanism. Indeed, TUS increased the median survival by 2.0 to 4.1 fold in both subcutaneous and orthotopic xenograft models.

The pharmacokinetic and toxicology studies revealed important favorable features of TUS. It is readily absorbed and achieves plasma concentrations sufficient to inhibit the set of key kinases, it has a plasma half-life that supports once daily dosing, and it demonstrated no adverse events of concerns in the toxicology studies. Although *in vitro* metabolic studies suggest that TUS may be metabolized by certain cytochrome P450 enzymes, observations in serum-free *in vitro* studies are unlikely to reflect significant relevance in the *in vivo* setting.

VEN increases the efficacy of many kinase inhibitors both *in vitro* and *in vivo* (16, 17, 25), and this proved to be the case for TUS as well. *In vitro* analysis of the interaction of TUS and VEN at the cellular level indicated an additive to a slightly synergistic level of enhancement. Yet, *in vivo,* the combination of these two drugs yielded a substantial improvement in therapeutic efficacy in the MOLM-14 FLT3-ITD xenograft model, and the benefit of combining TUS with VEN was greater than the level of benefit observed with the combination of gilteritinib with VEN. The combination of TUS and AZA was also more effective than either drug alone. All combinations were well tolerated. These results provide a strong rationale for clinical trials of the combination of TUS with either VEN or AZA or both.

Acquired resistance is a problem for AML therapeutic agents (21, 22). We established four MOLM-14 sublines that acquired resistance during prolonged continuous exposure to TUS. These lines were cross-resistant to gilteritinib but not to quizartinib indicating that the changes leading to TUS resistance did not reduce the dependence on FLT3-ITD-driven survival pathways. No new mutations were found in the FLT-ITD sequence already present in the parental cells, but all four resistant clones contained a new NRAS^G12C^ mutation and mutations in genes in which association with resistant to FLT3 inhibitors is largely unknown. Given that NRAS mutations are commonly found in patients whose AML is resistant to kinase inhibitors (8), and the demonstration that high level expression of NRAS^G12C^ in MOLM-14 cells yielded resistance to TUS, it seems likely that this mutation also mediates resistance.

Although TUS acutely reduced the phosphorylation levels of FLT3, SYK, STAT5, and ERK1/2 in the WT MOLM-14 cells, the phosphorylation levels of all of these were increased when the resistant cells were growing in 75 nmol/L TUS consistent with the concept that it requires activation of myriad bypass pathways in order to offset the cytotoxic effect of TUS. However, with the exception of slight hypersensitivity to the MEK inhibitor trametinib, the TUS/R cells were not more sensitive to fostamatinib (SYK inhibitor), ruxolitinib (JAK inhibitor), or dactolisib (PI3K inhibitor) as might be expected.

The most notable observation was the marked and unexpected synthetic lethal vulnerability to VEN and two MCL1 inhibitors in the TUS/R cells. In parental MOLM-14 cells exposed to TUS for 48 hours, the main perturbation of the pro- and antiapoptotic proteins was significant reduction in MCL1 and moderate increase in BIM. The BCL2 family proteins including MCL1 play a key role in regulation of cellular fate during normal and pathologic conditions (26). Multiple kinases and transcription factors have been demonstrated to play a key role in downregulation of MCL1 that is observed during apoptosis induction by FLT3 inhibitors (27). Upregulation of MCL1 is associated with drug resistance (28) and, consistent with prior reports, the TUS/R state was characterized by a modest decrease in BCL2 and no change in MCL1, but a significant increase in BAX suggesting a shift toward a more pro-apoptotic state consistent with recent studies of the determinants of sensitivity to VEN (29). The emergence of synthetic lethal hypersensitivity to VEN and the MCL1 inhibitors during treatment with TUS provides a very strong rationale for concurrent treatment with TUS and one of these drugs such that cells in which TUS resistant emerges may be readily killed by the co-administered drug.

Introduction of NRAS^G12D^ into MV-4-11 cells demonstrated that the level of expression of the mutant protein was more important than the presence or absence of the mutation. Low levels of expression did not cause resistance to TUS or VEN and actually rendered the cells more sensitive to the Raf, MEK, and MCL1 inhibitors tested. High levels of the NRAS^G12D^ protein were required to generate any measurable resistance to TUS, VEN, gilteritinib, and quizartinib. Hypersensitivity to the Raf inhibitor belvarafenib is of particular interest as the combination of TUS and belvarafenib was very effective *in vivo*. However, the effect of overexpression of NRAS^G12D^ from a strong promoter as in the MV-4-11 cells may differ from those produced when the gene is expressed from its endogenous promoter. The confounding effect of varying NRAS^G12D^ levels in different clones likely contributes to the heterogeneity of responsiveness among patients with AML identified with the NRAS^G12D^ mutation.

In summary, TUS is a well-differentiated kinase inhibitor with the advantage of targeting a spectrum of kinases known to be supportive of leukemic cell survival. TUS has favorable pharmacology and toxicology profiles with good oral absorption. Myelosuppression has not been observed in patients with relapsed/refractory AML who responded to treatment even with continuous and long-term dosing (30). TUS is highly active in orthotopic xenograft models of human AML and is well tolerated and more effective when combined with either VEN or AZA. Although the mechanisms underlying acquired resistance to TUS and the accompanying synthetic lethal vulnerability to VEN require further investigation, the findings provide a unique rationale for clinical investigation of the concurrent administration of drug combinations. These nonclinical findings suggest that TUS will demonstrate a favorable safety profile and a breadth of antileukemic activity across a breadth of patients with AML with a diversity of adverse mutations. A phase I/II trial of TUS/VEN in refractory patients and a separate phase I/II trial of the TUS/VEN/AZA triplet is underway in patients newly diagnosed AML (NCT03850574).

## Supplementary Material

Suppl Figure 1 Supplementary Figure 1

Suppl Figure 2 Supplementary Figure 2

Suppl Figure 3 Supplementary Figure 3

Suppl Figure 4 Supplementary Figure 4

Suppl Figure 5 Supplementary Figure 5

Suppl Figure 6 Supplementary Figure 6

Suppl Figure 7 Supplementary Figure 7

Suppl Figure 8 Supplementary Figure 8

Supplementary Tables Supplementary Table file to be published with manuscript

Supplementary Data Information on antibody RRID and Other catalog numbers
