# Supplementary material for: Preclinical Development of Tuspetinib for the Treatment of Acute Myeloid Leukemia
Source: Cancer Res Commun. 2025 Jan 13;5(1):74–83. doi: 10.1158/2767-9764.CRC-24-0258 (PMC11725774; doi:10.1158/2767-9764.CRC-24-0258)
Supplement: Suppl Figure 1 — Supplementary Figure 1 [file crc-24-0258_suppl_figure_1_suppsf1.pptx]

## Slide 1
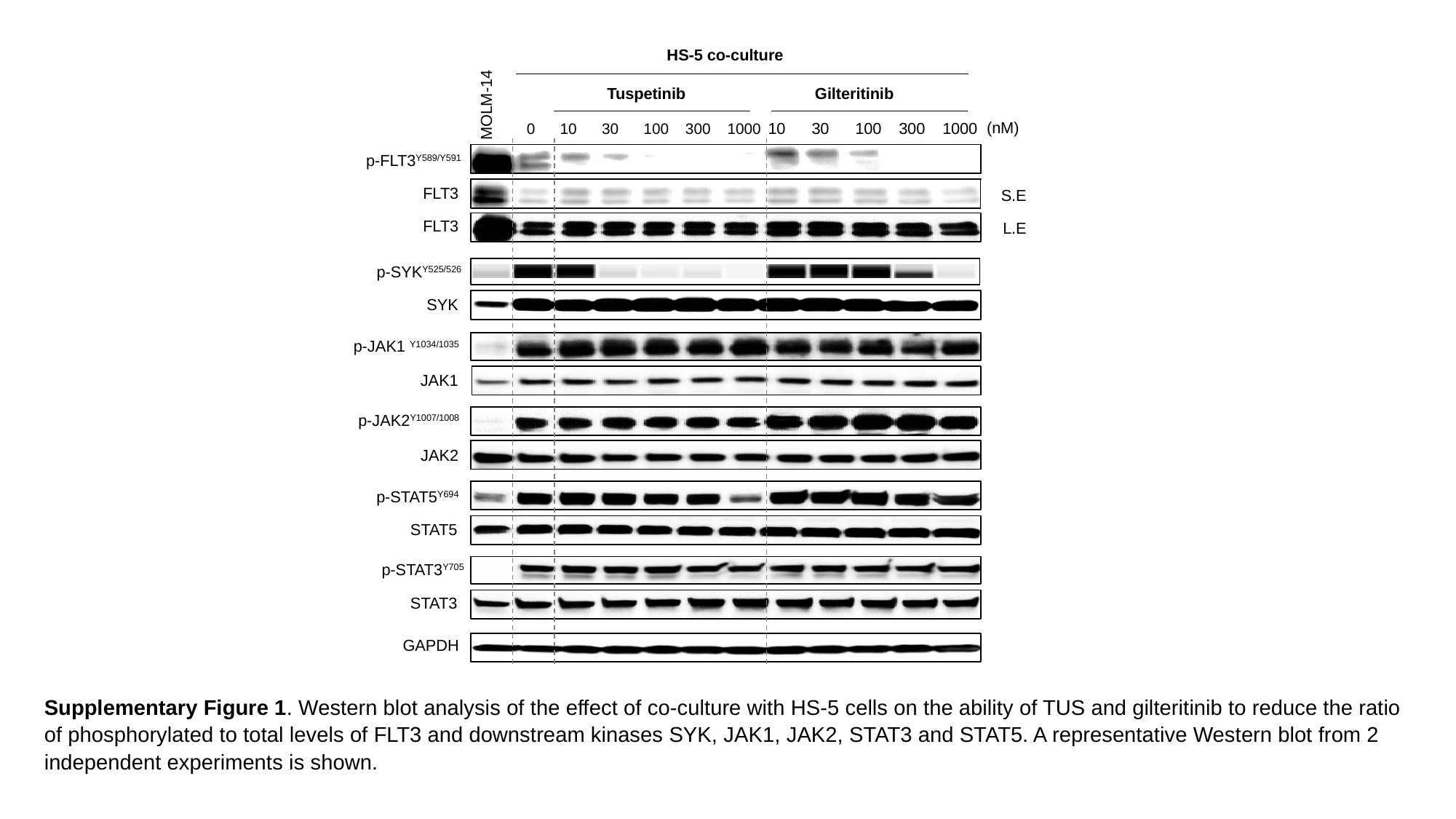

HS-5 co-culture
Gilteritinib
Tuspetinib
MOLM-14
 (nM)
10 30 100 300 1000
0 10 30 100 300 1000
p-FLT3Y589/Y591
FLT3
S.E
FLT3
L.E
p-SYKY525/526
SYK
p-JAK1 Y1034/1035
JAK1
p-JAK2Y1007/1008
JAK2
p-STAT5Y694
STAT5
p-STAT3Y705
STAT3
GAPDH
Supplementary Figure 1. Western blot analysis of the effect of co-culture with HS-5 cells on the ability of TUS and gilteritinib to reduce the ratio of phosphorylated to total levels of FLT3 and downstream kinases SYK, JAK1, JAK2, STAT3 and STAT5. A representative Western blot from 2 independent experiments is shown.
