# Supplementary material for: Preclinical Development of Tuspetinib for the Treatment of Acute Myeloid Leukemia
Source: Cancer Res Commun. 2025 Jan 13;5(1):74–83. doi: 10.1158/2767-9764.CRC-24-0258 (PMC11725774; doi:10.1158/2767-9764.CRC-24-0258)
Supplement: Suppl Figure 2 — Supplementary Figure 2 [file crc-24-0258_suppl_figure_2_suppsf2.pptx]

## Slide 1
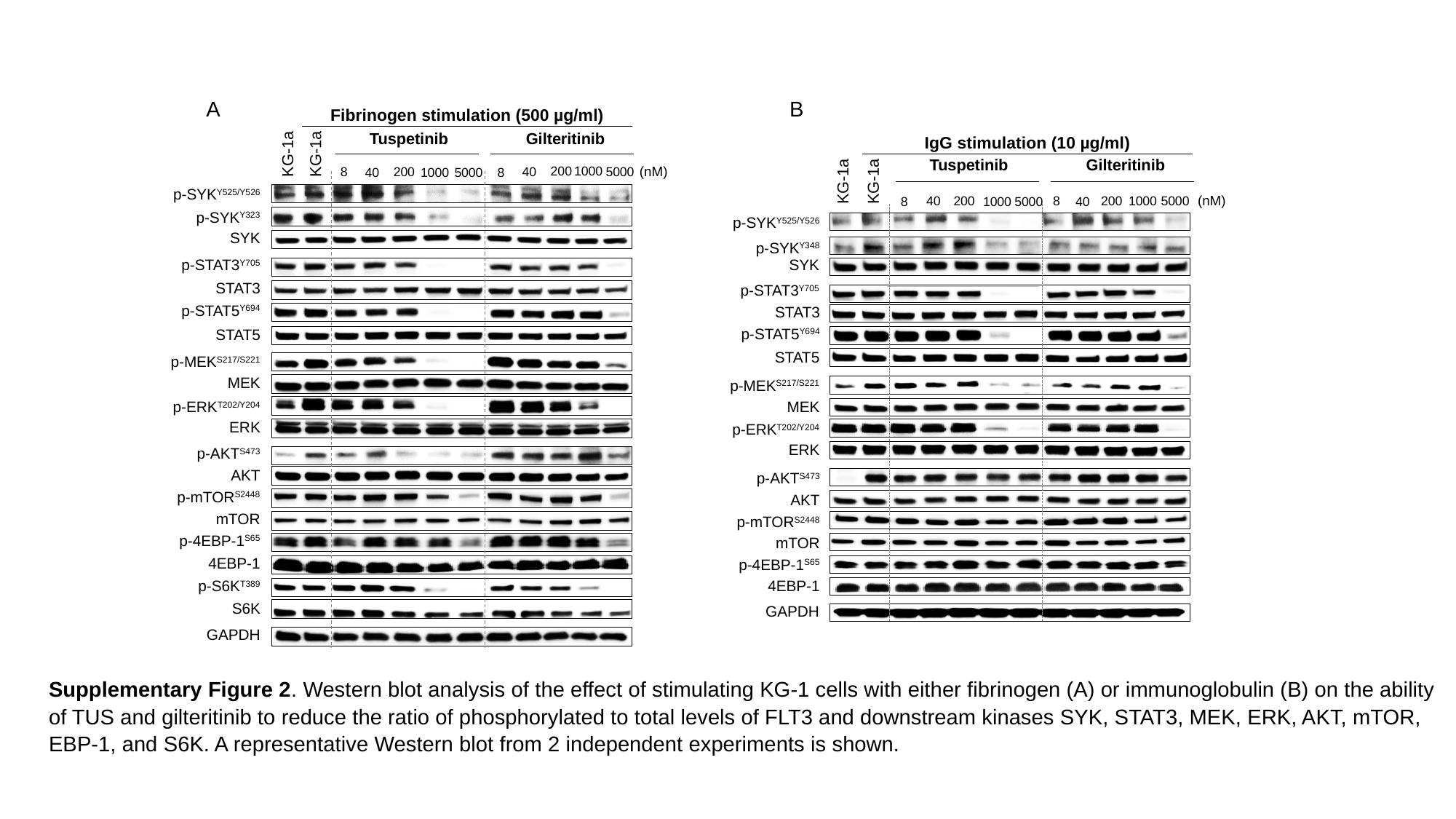

A
B
Fibrinogen stimulation (500 µg/ml)
Gilteritinib
Tuspetinib
KG-1a
KG-1a
(nM)
200
1000
5000
8
200
40
40
1000
5000
8
p-SYKY525/Y526
p-SYKY323
SYK
p-STAT3Y705
STAT3
p-STAT5Y694
STAT5
p-MEKS217/S221
MEK
p-ERKT202/Y204
ERK
p-AKTS473
AKT
p-mTORS2448
mTOR
p-4EBP-1S65
4EBP-1
p-S6KT389
S6K
GAPDH
IgG stimulation (10 µg/ml)
Tuspetinib
Gilteritinib
KG-1a
KG-1a
8
(nM)
40
200
1000
200
5000
8
1000
40
5000
p-SYKY525/Y526
p-SYKY348
SYK
p-STAT3Y705
STAT3
p-STAT5Y694
STAT5
p-MEKS217/S221
MEK
p-ERKT202/Y204
ERK
p-AKTS473
AKT
p-mTORS2448
mTOR
p-4EBP-1S65
4EBP-1
GAPDH
Supplementary Figure 2. Western blot analysis of the effect of stimulating KG-1 cells with either fibrinogen (A) or immunoglobulin (B) on the ability of TUS and gilteritinib to reduce the ratio of phosphorylated to total levels of FLT3 and downstream kinases SYK, STAT3, MEK, ERK, AKT, mTOR, EBP-1, and S6K. A representative Western blot from 2 independent experiments is shown.
