# Supplementary material for: Preclinical Development of Tuspetinib for the Treatment of Acute Myeloid Leukemia
Source: Cancer Res Commun. 2025 Jan 13;5(1):74–83. doi: 10.1158/2767-9764.CRC-24-0258 (PMC11725774; doi:10.1158/2767-9764.CRC-24-0258)
Supplement: Suppl Figure 3 — Supplementary Figure 3 [file crc-24-0258_suppl_figure_3_suppsf3.pptx]

## Slide 1
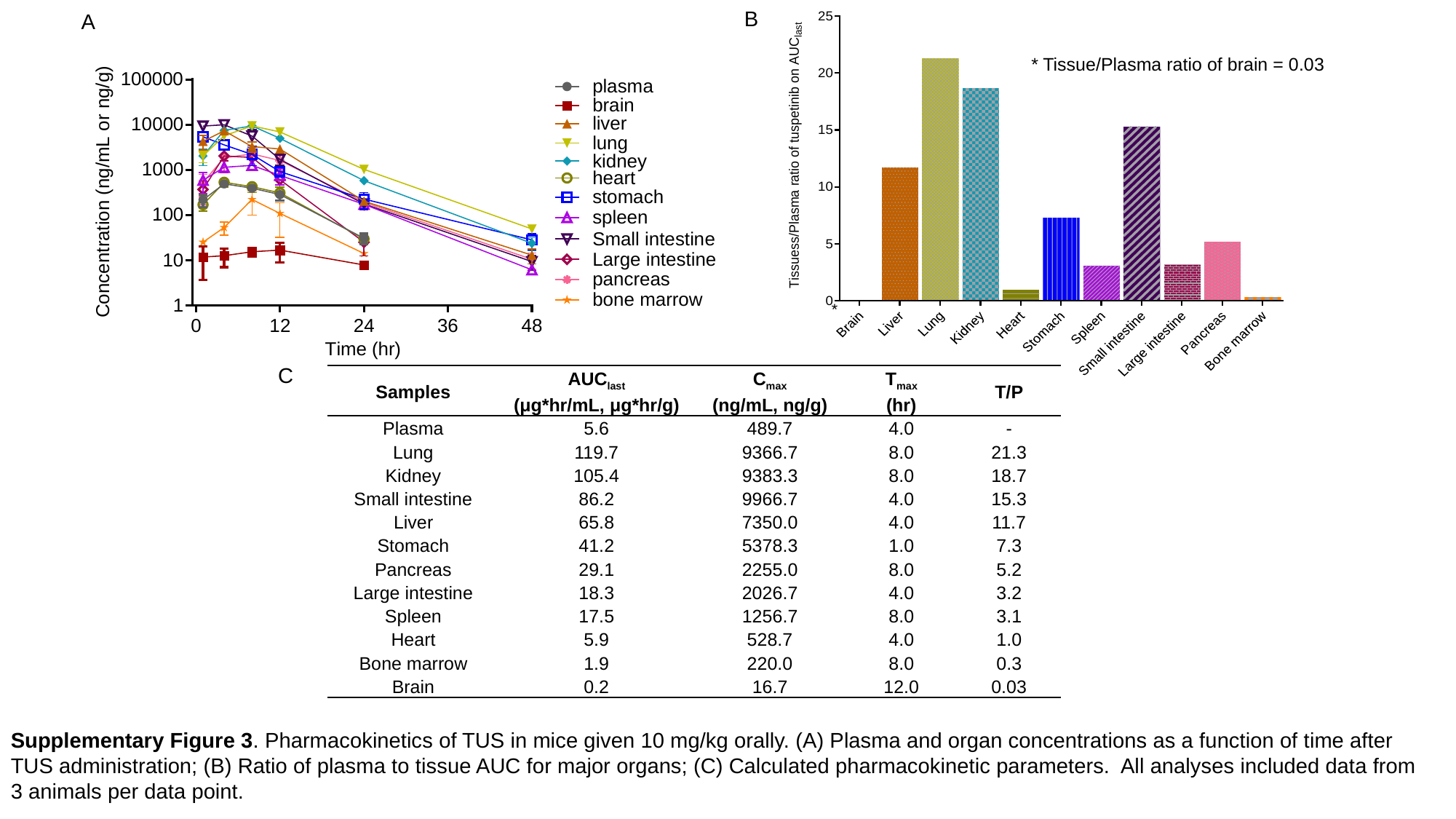

B
A
* Tissue/Plasma ratio of brain = 0.03
C
| Samples | AUClast (μg\*hr/mL, μg\*hr/g) | Cmax (ng/mL, ng/g) | Tmax (hr) | T/P |
| --- | --- | --- | --- | --- |
| Plasma | 5.6 | 489.7 | 4.0 | - |
| Lung | 119.7 | 9366.7 | 8.0 | 21.3 |
| Kidney | 105.4 | 9383.3 | 8.0 | 18.7 |
| Small intestine | 86.2 | 9966.7 | 4.0 | 15.3 |
| Liver | 65.8 | 7350.0 | 4.0 | 11.7 |
| Stomach | 41.2 | 5378.3 | 1.0 | 7.3 |
| Pancreas | 29.1 | 2255.0 | 8.0 | 5.2 |
| Large intestine | 18.3 | 2026.7 | 4.0 | 3.2 |
| Spleen | 17.5 | 1256.7 | 8.0 | 3.1 |
| Heart | 5.9 | 528.7 | 4.0 | 1.0 |
| Bone marrow | 1.9 | 220.0 | 8.0 | 0.3 |
| Brain | 0.2 | 16.7 | 12.0 | 0.03 |
Supplementary Figure 3. Pharmacokinetics of TUS in mice given 10 mg/kg orally. (A) Plasma and organ concentrations as a function of time after TUS administration; (B) Ratio of plasma to tissue AUC for major organs; (C) Calculated pharmacokinetic parameters. All analyses included data from 3 animals per data point.
