# Supplementary material for: Preclinical Development of Tuspetinib for the Treatment of Acute Myeloid Leukemia
Source: Cancer Res Commun. 2025 Jan 13;5(1):74–83. doi: 10.1158/2767-9764.CRC-24-0258 (PMC11725774; doi:10.1158/2767-9764.CRC-24-0258)
Supplement: Suppl Figure 4 — Supplementary Figure 4 [file crc-24-0258_suppl_figure_4_suppsf4.pptx]

## Slide 1
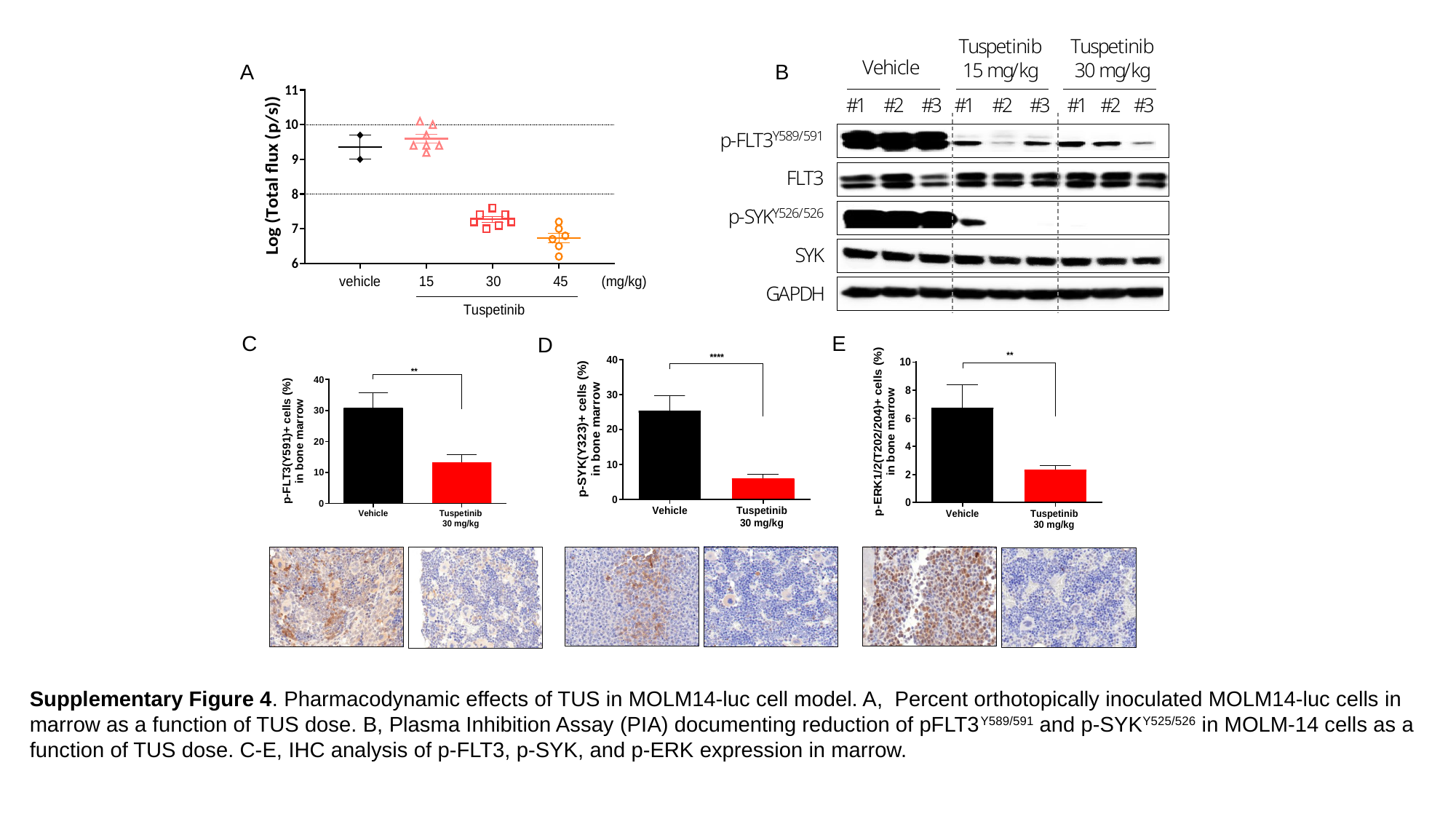

B
A
C
E
D
Supplementary Figure 4. Pharmacodynamic effects of TUS in MOLM14-luc cell model. A, Percent orthotopically inoculated MOLM14-luc cells in marrow as a function of TUS dose. B, Plasma Inhibition Assay (PIA) documenting reduction of pFLT3Y589/591 and p-SYKY525/526 in MOLM-14 cells as a function of TUS dose. C-E, IHC analysis of p-FLT3, p-SYK, and p-ERK expression in marrow.
