# Supplementary material for: Preclinical Development of Tuspetinib for the Treatment of Acute Myeloid Leukemia
Source: Cancer Res Commun. 2025 Jan 13;5(1):74–83. doi: 10.1158/2767-9764.CRC-24-0258 (PMC11725774; doi:10.1158/2767-9764.CRC-24-0258)
Supplement: Suppl Figure 5 — Supplementary Figure 5 [file crc-24-0258_suppl_figure_5_suppsf5.pptx]

## Slide 1
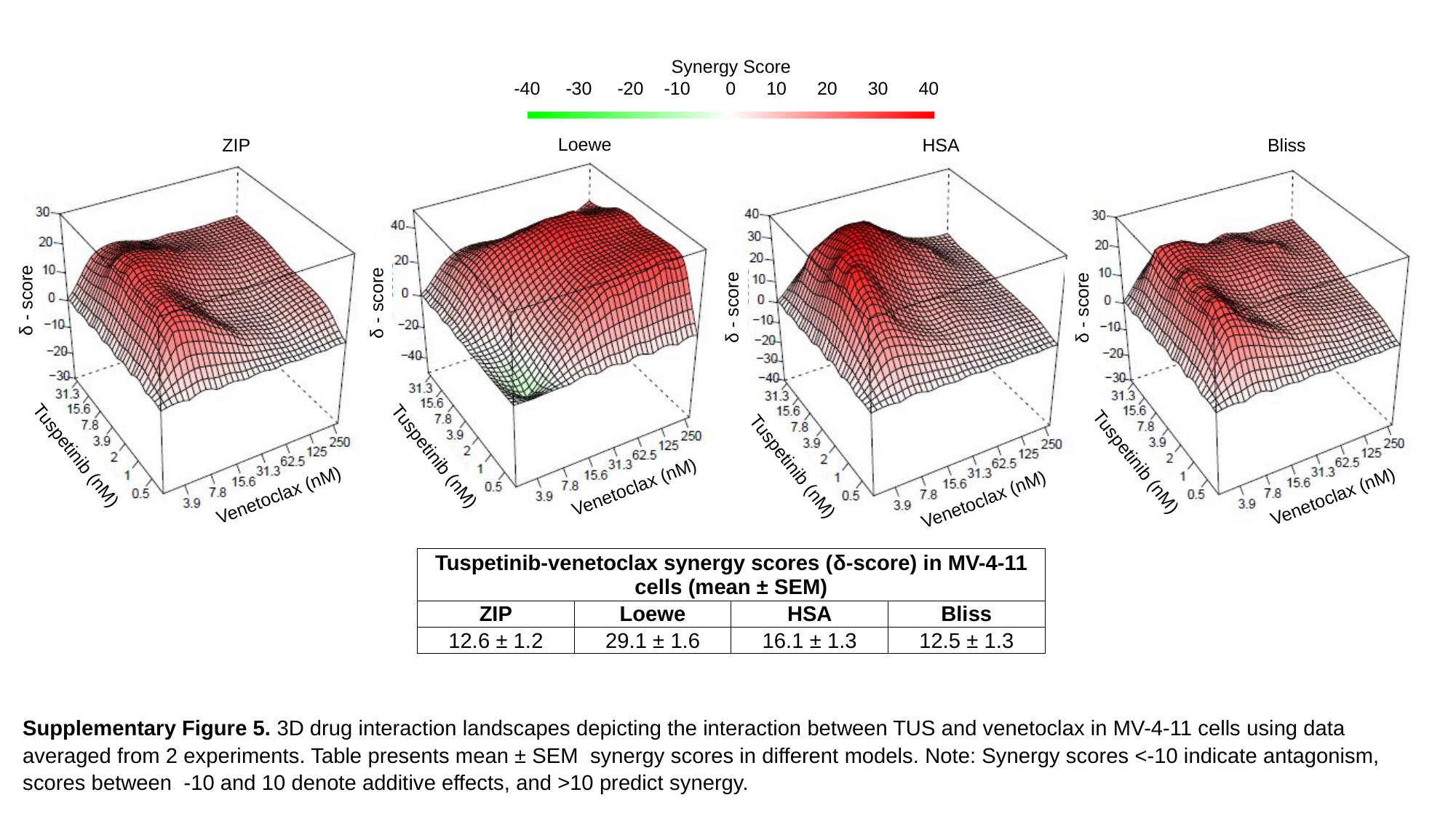

Synergy Score
-40 -30 -20 -10 0 10 20 30 40
Loewe
δ - score
Tuspetinib (nM)
Venetoclax (nM)
ZIP
δ - score
Tuspetinib (nM)
Venetoclax (nM)
HSA
δ - score
Tuspetinib (nM)
Venetoclax (nM)
Bliss
δ - score
Tuspetinib (nM)
Venetoclax (nM)
| Tuspetinib-venetoclax synergy scores (δ-score) in MV-4-11 cells (mean ± SEM) | | | |
| --- | --- | --- | --- |
| ZIP | Loewe | HSA | Bliss |
| 12.6 ± 1.2 | 29.1 ± 1.6 | 16.1 ± 1.3 | 12.5 ± 1.3 |
Supplementary Figure 5. 3D drug interaction landscapes depicting the interaction between TUS and venetoclax in MV-4-11 cells using data averaged from 2 experiments. Table presents mean ± SEM synergy scores in different models. Note: Synergy scores <-10 indicate antagonism, scores between -10 and 10 denote additive effects, and >10 predict synergy.
