# Supplementary material for: Preclinical Development of Tuspetinib for the Treatment of Acute Myeloid Leukemia
Source: Cancer Res Commun. 2025 Jan 13;5(1):74–83. doi: 10.1158/2767-9764.CRC-24-0258 (PMC11725774; doi:10.1158/2767-9764.CRC-24-0258)
Supplement: Suppl Figure 6 — Supplementary Figure 6 [file crc-24-0258_suppl_figure_6_suppsf6.pptx]

## Slide 1
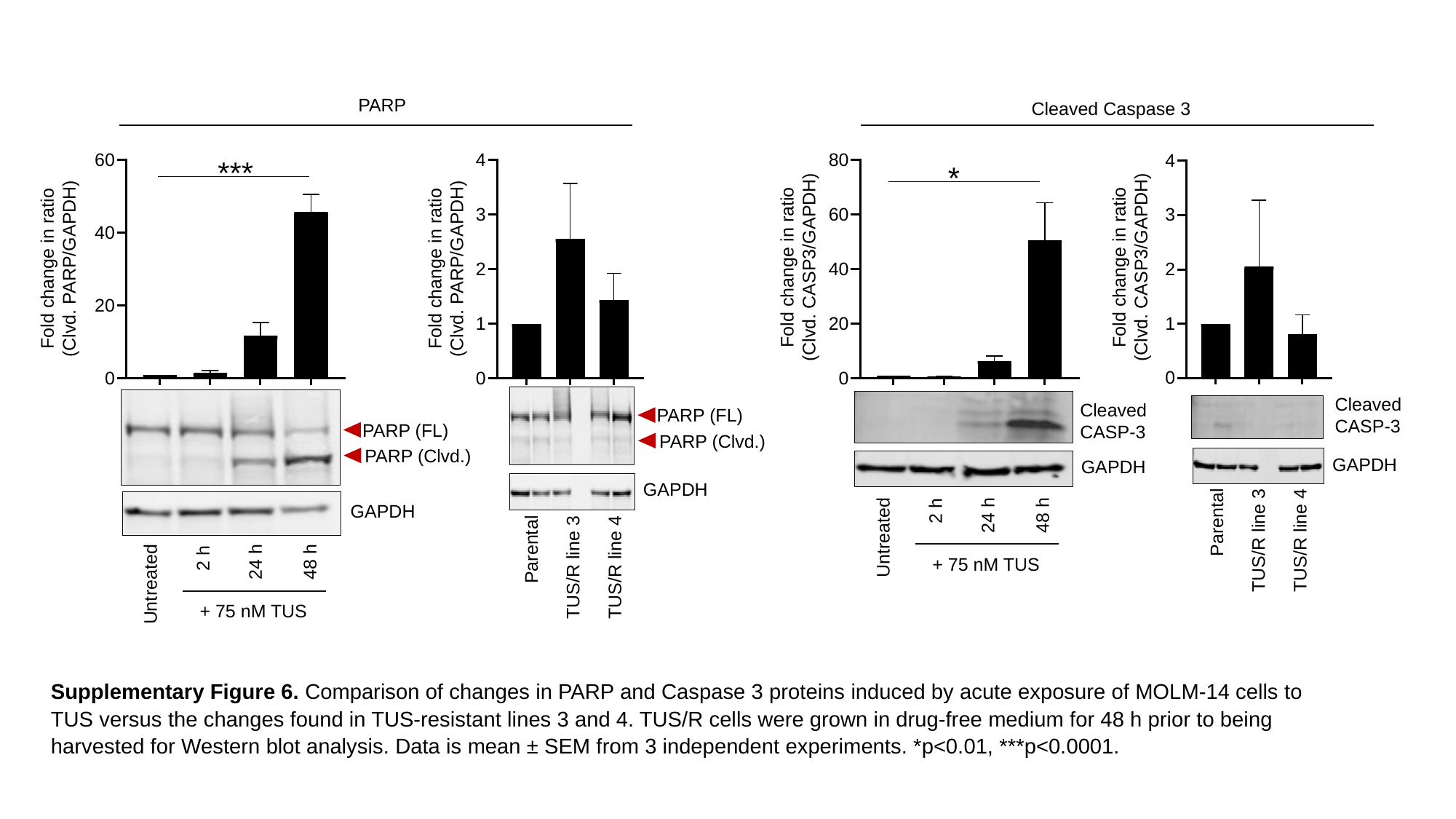

PARP
Fold change in ratio (Clvd. PARP/GAPDH)
PARP (FL)
PARP (Clvd.)
GAPDH
2 h
24 h
48 h
Untreated
+ 75 nM TUS
Fold change in ratio (Clvd. PARP/GAPDH)
PARP (FL)
PARP (Clvd.)
GAPDH
Parental
TUS/R line 3
TUS/R line 4
Cleaved Caspase 3
Fold change in ratio (Clvd. CASP3/GAPDH)
Cleaved
CASP-3
GAPDH
2 h
24 h
48 h
Untreated
+ 75 nM TUS
Fold change in ratio (Clvd. CASP3/GAPDH)
Cleaved
CASP-3
GAPDH
Parental
TUS/R line 3
TUS/R line 4
***
*
Supplementary Figure 6. Comparison of changes in PARP and Caspase 3 proteins induced by acute exposure of MOLM-14 cells to TUS versus the changes found in TUS-resistant lines 3 and 4. TUS/R cells were grown in drug-free medium for 48 h prior to being harvested for Western blot analysis. Data is mean ± SEM from 3 independent experiments. *p<0.01, ***p<0.0001.
