# Supplementary material for: Preclinical Development of Tuspetinib for the Treatment of Acute Myeloid Leukemia
Source: Cancer Res Commun. 2025 Jan 13;5(1):74–83. doi: 10.1158/2767-9764.CRC-24-0258 (PMC11725774; doi:10.1158/2767-9764.CRC-24-0258)
Supplement: Suppl Figure 7 — Supplementary Figure 7 [file crc-24-0258_suppl_figure_7_suppsf7.pptx]

## Slide 1
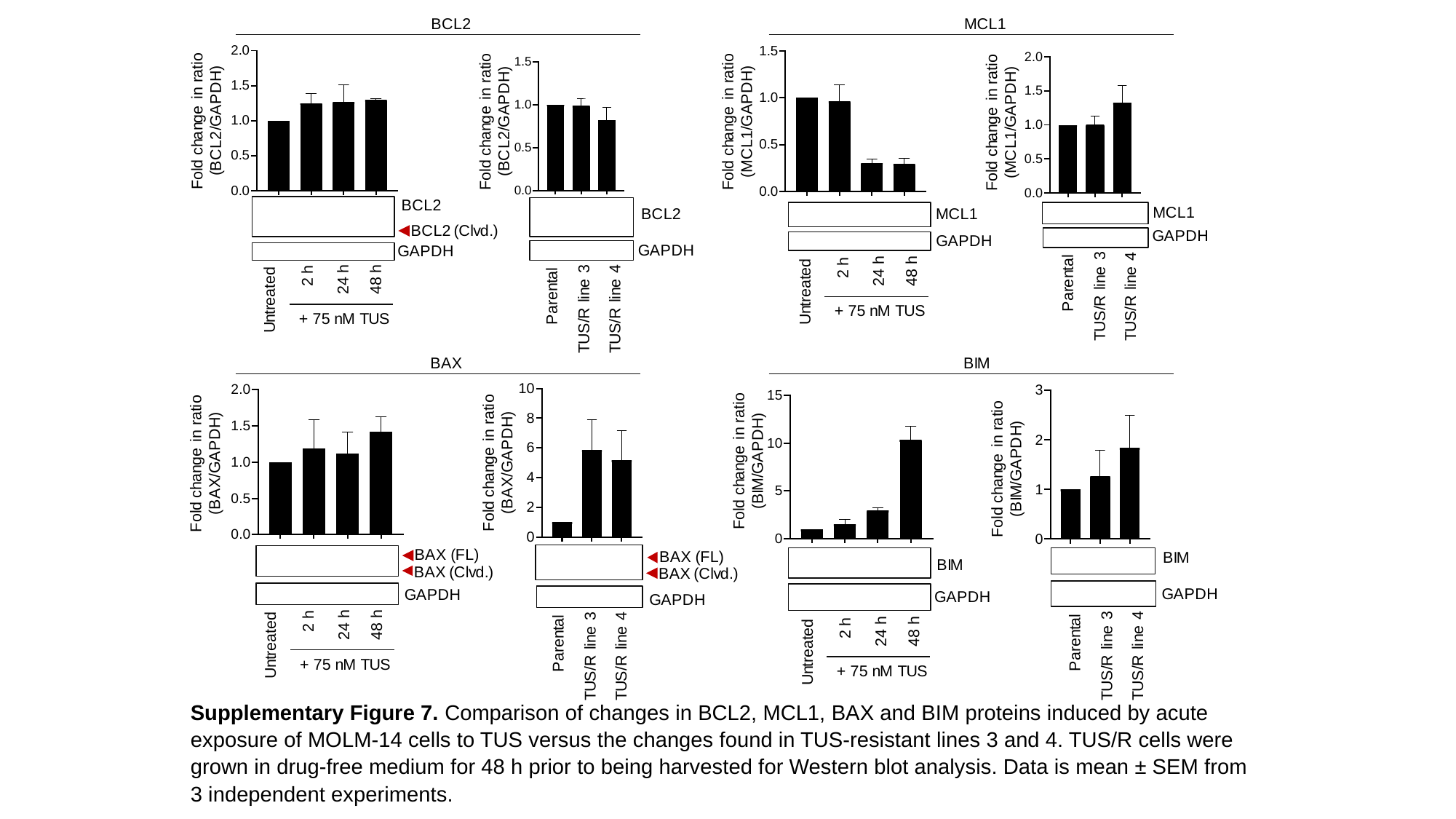

Supplementary Figure 7. Comparison of changes in BCL2, MCL1, BAX and BIM proteins induced by acute exposure of MOLM-14 cells to TUS versus the changes found in TUS-resistant lines 3 and 4. TUS/R cells were grown in drug-free medium for 48 h prior to being harvested for Western blot analysis. Data is mean ± SEM from 3 independent experiments.
