# Supplementary material for: Preclinical Development of Tuspetinib for the Treatment of Acute Myeloid Leukemia
Source: Cancer Res Commun. 2025 Jan 13;5(1):74–83. doi: 10.1158/2767-9764.CRC-24-0258 (PMC11725774; doi:10.1158/2767-9764.CRC-24-0258)
Supplement: Suppl Figure 8 — Supplementary Figure 8 [file crc-24-0258_suppl_figure_8_suppsf8.pptx]

## Slide 1
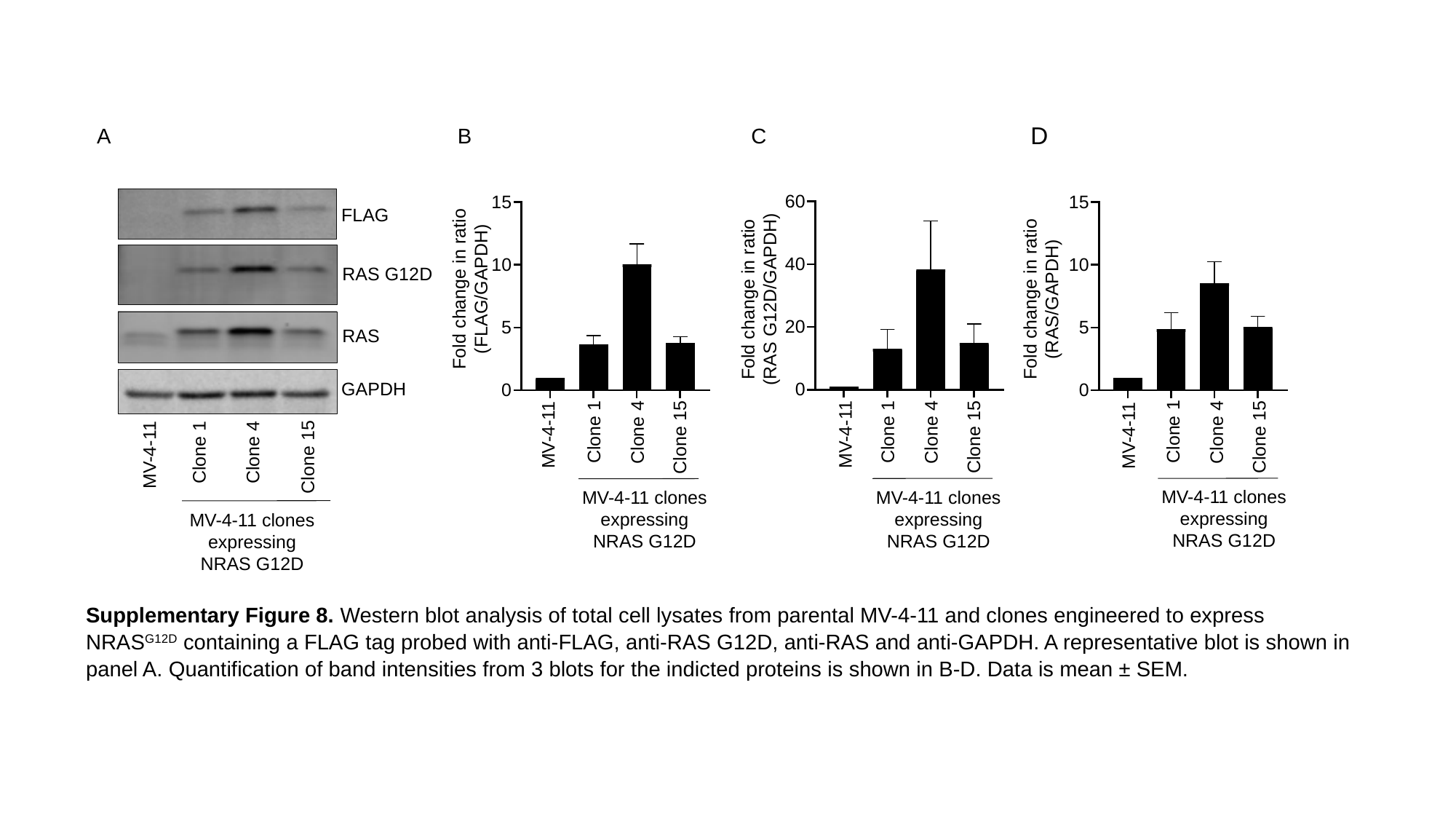

D
Fold change in ratio (RAS/GAPDH)
Clone 1
MV-4-11
Clone 4
Clone 15
MV-4-11 clones expressing NRAS G12D
A
FLAG
RAS G12D
RAS
GAPDH
Clone 1
Clone 4
MV-4-11
Clone 15
MV-4-11 clones expressing NRAS G12D
B
Fold change in ratio (FLAG/GAPDH)
Clone 1
Clone 4
MV-4-11
Clone 15
MV-4-11 clones expressing NRAS G12D
C
Fold change in ratio (RAS G12D/GAPDH)
Clone 1
MV-4-11
Clone 4
Clone 15
MV-4-11 clones expressing NRAS G12D
Supplementary Figure 8. Western blot analysis of total cell lysates from parental MV-4-11 and clones engineered to express NRASG12D containing a FLAG tag probed with anti-FLAG, anti-RAS G12D, anti-RAS and anti-GAPDH. A representative blot is shown in panel A. Quantification of band intensities from 3 blots for the indicted proteins is shown in B-D. Data is mean ± SEM.
