# Supplementary material for: Preclinical Development of Tuspetinib for the Treatment of Acute Myeloid Leukemia
Source: Cancer Res Commun. 2025 Jan 13;5(1):74–83. doi: 10.1158/2767-9764.CRC-24-0258 (PMC11725774; doi:10.1158/2767-9764.CRC-24-0258)
Supplement: Supplementary Tables — Supplementary Table file to be published with manuscript [file crc-24-0258_supplementary_tables_suppst.docx]

**Supplementary Tables**

| **Supplementary Table 1. Relative potency of FLT3 inhibitors** | | | |
| --- | --- | --- | --- |
| **Kinases** | **GI_50_ (nM)** | | |
|  | **TUS** | **Gilteritinib** | **Midostaurin** |
| FLT3 WT | 1.1 ± 0.2 | 3.0 ± 0.3 | 2.9 ± 0.5 |
| FLT3 ITD | 1.8 ± 0.2 | 0.80 ± 0.3 | 2.9 ± 0.8 |
| FLT3 D835Y | 1.0 ± 0.1 | 0.5 ± 0.1 | 2.2 ± 0.5 |
| Data is mean ± SD. | | | |

| **Supplementary Table 2.** Binding affinity of TUS to wild type and mutant forms of FLT3. | | | | |
| --- | --- | --- | --- | --- |
| **Kinases** | **K_d_ (nM)** | | | |
|  | **TUS** | **Gilteritinib** | **Quizartinib** | **Midostaurin** |
| FLT3 WT | 0.6 ± 0.2 | 4.4 ± 1.1 | 4.9 ± 1.2 | 6.1 ± 2.5 |
| FLT3 ITD | 0.4 ± 0.2 | 1.0 ± 0.5 | 9.5 ± 0.2 | 2.4 ± 0.3 |
| FLT3 D835H | 0.4 ± 0.1 | 1.5 ± 0.6 | 3.0 ± 0.1 | 1.4 ± 0.4 |
| FLT3 D835V | 0.5 ± 0.5 | 0.3 ± 0.1 | 20 ± 16 | 4.5 ± 3.7 |
| FLT3 D835Y | 0.3 ± 0.04 | 0.8 ± 0.6 | 11 ± 2.1 | 1.3 ± 0.2 |
| FLT3 K663Q | 0.3 ± 0.2 | 15 ± 11 | 1.8 ± 0.2 | 2.3 ± 1.5 |
| FLT3 N841I | 0.2 ± 0.1 | 7.2 ± 3.8 | 2.6 ± 1.6 | 1.6 ± 1.1 |
| FLT3 ITD/D835V | 0.5 ± 0.01 | 0.2 ± 0.02 | > 80 | 5.8 ± 1.9 |
| FLT3 ITD/F691L | 1.3 ± 0.4 | 0.4 ± 0.1 | > 100 | 9.3 ± 5.2 |
| Data is mean ± SD. | | | | |

| **Supplementary Table 3**. Potency of TUS in Ba/F3 cells expressing WT and mutant forms of FLT3. | | | | |
| --- | --- | --- | --- | --- |
| **Ba/F3 cell lines** | **GI_50_ (nM)** | | | |
|  | **TUS** | **Gilteritinib** | **Quizartinib** | **Midostaurin** |
| FLT3 WT | 9.1 ± 2.0 | 7.0 ± 1.5 | 2.5 ± 0.8 | 22 ± 8.0 |
| FLT3 ITD | 2.5 ± 0.4 | 1.2 ± 0.2 | 0.4 ± 0.1 | 5.1 ± 0.1 |
| FLT3 ITD/F691L | 56 ± 13 | 33 ± 6.2 | 131 ± 33 | 16 ± 3.5 |
| FLT3 D835Y | 7.3 ± 3.5 | 2.3 ± 0.7 | 14 ± 2.6 | 9.0 ± 2.7 |
| FLT3 ITD/D835Y | 16 ± 1.7 | 4.4 ± 0.6 | 103 ± 6.8 | 21 ± 1.1 |
| Data is mean ± SD. | | | | |

| **Supplementary Table 4.** Potency of TUS in AML cell lines. | | | | |
| --- | --- | --- | --- | --- |
| **Cell lines** | **GI_50_ (nM)** | | | |
|  | **TUS** | **Gilteritinib** | **Quizartinib** | **Midostaurin** |
| MV-4-11 | 1.3 ± 0.2 | 2.0 ± 0.2 | 0.2 ± 0.1 | 6.0 ± 1.4 |
| MOLM-13 | 5.2 ± 0.9 | 8.4 ± 2.9 | 0.9 ± 0.5 | 19 ± 14 |
| MOLM-14 | 2.9 ± 1.2 | 6.1 ± 2.8 | 0.6 ± 0.2 | 12 ± 7.1 |
| Data is mean ± SD. | | | | |

| **Supplementary Table 5.** Additional genes in which mutations were found in TUS/R clones but not found in the parental MOLM-14 cells. |
| --- |
| BRD9, CCDC74A, CDT1, CES1, CILP, DTX4, EML2, FAT3, FGFBP1, FNDC7, GPRIN1, H3-3A, HHIP, HLA-DQB2, HS6ST1, JARID2, LIFR, LRRC14B, MAJIN, MMP19, NECAB1, NKTR, OR10H1, POU2AF3, RAD51D, RBMX, SLC47A2, SLITRK4, SPTA1, SURF1, TARP, THSD4, TMEM198B, TRIM36, TRPS1, TTF2, ZBTB10, ZIK1. |

| **Supplementary Table 6.** Table showing IC_50_ of parental MOLM-14 and TUS/R cells against inhibitors of some selected targets. | | | | |
| --- | --- | --- | --- | --- |
| **Drug** | **Predicted target (s)** | **GI_50_ (nM)** | | |
|  |  | **Parental MOLM-14** | **TUS/R line 3** | **TUS/R line 4** |
| Tuspetinib  Gilteritinib | Multi-kinase  FLT3 | 2.3 ± 0.6  8.6 ± 0.2 | 108.0 ± 24**  118.4 ± 8.5** | 144.4 ± 31**  128.1 ± 8.9** |
| Quizartinib | FLT3 | 0.5 ± 0.02 | 0.2 ± 0.1^#^ | 0.5 ± 0.1^ns^ |
| Fostamatinib | SYK | 99.6. ± 30.8 | 175.7 ± 32.9^ns^ | 459.3 ± 86.2^ns^ |
| Ruxolitinib | JAK1, JAK2 | ≈8254 | ≈1100.6 | ≈1125.4 |
| Dactolisib | PI3K | 21.3 ± 1.4 | 28.9 ± 2.7^ns^ | 26.8 ± 1.8^ns^ |
| Trametinib | MEK | 153.4 ± 14.5 | 67.0 ± 12.8^#^ | 101.5 ± 0.4^#^ |
| 5-Azacytidine | DNMTi | 2137.7 ± 231.2 | 1615.3 ± 294.5^ns^ | 1580.7 ± 145.2^ns^ |
| Luxeptinib | Pan-FLT3, Pan-BTK | 0.2 ± 0.01 | 0.1 ± 0.01** | 0.1 ± 0.01** |
| Obatoclax | BCL-2, BCL-w, BCL-xL, MCL1 | 98.2 ± 7.7 | 164.3 ± 14.7^#^ | 142.1 ± 9.1^#^ |
| Navitoclax | BCL2 | 409.6 ± 50.2 | 15.3 ± 2.4** | 15.0 ± 2.1** |
| AZD 5991 | MCL1 | 167.9 ± 33.7 | 22.8 ± 2.7* | 18.2 ± 1.6* |
| S63845 | MCL1 | 105.3 ± 19.8 | 5.6 ± 0.7* | 4.6 ± 0.3* |
| Data is mean ± SEM. *p<0.01, **p<0.001, #p<0.05, ns = not significant vs MOLM-14 parental cells. | | | | |

| **Supplementary Table 7.** IC_50_ values (nM) of drugs against parental MV-4-11 and MV-4-11 engineered to express NRAS G12D Clones 1, 4 and 15. | | | | |
| --- | --- | --- | --- | --- |
| **Drug** | **GI_50_ (nM)** | | | |
|  | **Parental (MV-4-11)** | **Clone 1** | **Clone 4** | **Clone 15** |
| TUS | 9.1 ± 2.3 | 8.7 ± 3.0 | 69.9 ± 7.4** | 7.7 ± 3.1 |
| Venetoclax | 10.5 ± 1.7 | 5.5 ± 1.4 | 2256.0 ± 156.2*** | 4.9 ± 1.3 |
| Belvarafenib | 750.0 ± 92.8 | 186.7 ± 38.6** | 209.3 ± 33.9** | 104.1 ± 11.1** |
| Trametinib | 6.7 ± 1.6 | 1.6 ± 0.4 | 2.5 ± 0.7 | 2.3 ± 0.8 |
| S63845 | 4.6 ± 0.4 | 1.1 ± 0.3** | 7.3 ± 1.5 | 1.5 ± 0.1** |
| AZD5991 | 32.5 ± 5.4 | 10.1 ± 3.2^#^ | 44.3 ± 8.3 | 7.2 ± 0.4 |
| Gilteritinib | 2.4 ± 0.7 | 3.3 ± 0.7 | 51.0 ± 12.2* | 5.6 ± 1.1 |
| Quizartinib | 0.5 ± 0.1 | 1.1 ± 0.2^ns^ | 1.9 ± 0.5^ns^ | 1.3 ± 0.4^ns^ |
| Data is mean ± SEM. *p<0.01, **p<0.001, #p = 0.02, ns = not significant vs parental MV-4-11 cells. | | | | |

| **Supplementary Table 8.** Table showing synergy scores of TUS combined with either belvarafenib, S63845 or venetoclax in MV-4-11 cells engineered to express NRAS G12D Clone 4 cells calculated using ZIP, Loewe, HSA or Bliss algorithms. | | | | |
| --- | --- | --- | --- | --- |
| **Drug combination** | **Synergy Scores** | | | |
|  | **ZIP** | **Loewe** | **HSA** | **Bliss** |
| TUS + Belvarafenib | 12.5 ± 1.1 | 31.3 ± 2.9 | 19.8 ± 1.1 | 12.5 ± 1.1 |
| TUS + S63845 | 10.5 ± 1.2 | 6.3 ± 2.9 | 14.7 ± 1.4 | 10.5 ± 1.2 |
| TUS + Venetoclax | 11.9 ± 0.9 | 19.9 ± 1.6 | 17.2 ± 1.3 | 11.7 ± 1.1 |
| Data is mean ± SEM. | | | | |
